# Supplementary material for: Cryptic Species Exist in Vietnamella sinensis Hsu, 1936 (Insecta: Ephemeroptera) from Studies of Complete Mitochondrial Genomes
Source: Insects. 2022 Apr 26;13(5):412. doi: 10.3390/insects13050412 (PMC9143467; doi:10.3390/insects13050412)
Supplement: Supplementary file 1 [file insects-13-00412-s001.zip › TableS2. IGNs.pdf]

Table S2. The size variation between *V. sinensis* CN/TL (CN/TL), *V. sinensis* QY (QY) and *V. sinensis* (VS) (HM067837).

tRNA/IGN	CN/TL	QY	VS (HM067837)	tRNA/IGN	CN/TL	QY	VS (HM067837)
$tRNA^{Ile}$	64	65	64	$tRNA^{Asn}$	64	64	64
IGN	(+)11	(+)14	(+)7	IGN	(-)3	(-)3	(-)4
$tRNA^{Gln}$	69	69	69	$tRNA^{Ser1}$	66	66	68
IGN	(+)18	(+)16	(+)19	IGN	0	0	(-)1
$tRNA^{Met}$	64	64	64	$tRNA^{Glu}$	63	63	63
IGN	0	0	0	IGN	(-)2	(+)25	(-)2
ND2	1023	1023	1023	$tRNA^{Phe}$	63	64	63
IGN	(-)2	(-)2	(-)2	IGN	0	0	0
$tRNA^{Trp}$	69	69	69	ND5	1729	1729	1729
IGN	(-)8	(-)8	(-)8	IGN	0	0	0
$tRNA^{Cys}$	61	61	61	$tRNA^{His}$	62	62	63
IGN	0	0	0	IGN	0	0	0
$tRNA^{Tyr}$	70	70	70	ND4	1347	1347	1347
IGN	(-)41	(-)41	(-)41	IGN	(-)7	(-)7	(-)7
COI	1578	1578	1578	ND4L	297	297	294
IGN	(-)5	(-)5	(-)5	IGN	(+)2	(+)2	(+)2
$tRNA^{Leu2}$	64	64	64	$tRNA^{Thr}$	62	62	62
IGN	0	0	0	IGN	0	0	0
COII	688	688	688	$tRNA^{Pro}$	64	64	64
IGN	0	0	0	IGN	(+)5	(+)5	(+)5
$tRNA^{Lys}$	69	69	70	ND6	516	516	516
IGN	0	0	(-)1	IGN	(-)1	(-)1	(-)1
$tRNA^{Asp}$	67	67	67	Cyt *b*	1135	1135	1135
IGN	(+)9	(+)9	(+)9	IGN	0	0	0
ATP8	156	156	156	$tRNA^{Ser2}$	67	67	67
IGN	(-)4	(-)4	(-)4	IGN	(+)18	(+)30	(-)20
ATP6	675	675	675	ND1	939	939	939
IGN	(-)1	(-)1	(-)1	IGN	(+)1	(+)1	(+)1
COIII	789	789	789	$tRNA^{Leu1}$	64	65	64
IGN	(+)4	(+)4	(+)4	IGN	0	0	0
$tRNA^{Gly}$	62	62	62	16S rRNA	1223	1221	1251
IGN	(-)3	(-)3	(-)3	IGN	0	0	(-)28
ND3	357	357	357	$tRNA^{Val}$	66	66	66
IGN	(-)2	(-)2	(-)2	IGN	0	0	0
$tRNA^{Ala}$	65	66	67	12S rRNA	792	790	793
IGN	0	0	(-)1	IGN	0	0	0
$tRNA^{Arg}$	64	64	63	CR	1015	911	1105
IGN	(-)3	(-)3	(-)3				
