# Supplementary material for: Cryptic Species Exist in Vietnamella sinensis Hsu, 1936 (Insecta: Ephemeroptera) from Studies of Complete Mitochondrial Genomes
Source: Insects. 2022 Apr 26;13(5):412. doi: 10.3390/insects13050412 (PMC9143467; doi:10.3390/insects13050412)
Supplement: Supplementary file 1 [file insects-13-00412-s001.zip › TableS3. (TL+CN) location.pdf]

Table S3. Location of features in the mtDNA of *V. sinensis* CN/TL.

Gene	Strand	Position	Length (nuc.)	Anti Codon	Start Codon	Stop Codon	Intergenic nucleotides
$tRNA^{Ile}$	+	1-64	64	ATC			0
$tRNA^{Gln}$	-	76-144	69	CAA			+11
$tRNA^{Met}$	+	163-226	64	ATG			+18
ND2	+	227-1249	1023		ATA	TAA	0
$tRNA^{Trp}$	+	1248-1316	69	TGA			-2
$tRNA^{Cys}$	-	1309-1369	61	TGC			-8
$tRNA^{Tyr}$	-	1370-1439	70	TAC			0
COI	+	1399-2976	1578		ATA	TAA	-41
$tRNA^{Leu2}$	+	2972-3035	64	TAA			-5
COII	+	3036-3723	688		ATG	T	0
$tRNA^{Lys}$	+	3724-3792	69	AAG			0
$tRNA^{Asp}$	+	3793-3859	67	GAC			0
ATP8	+	3869-4024	156		ATA	TAA	+9
ATP6	+	4021-4695	675		ATA	TAA	-4
COIII	+	4695-5483	789		ATG	TAA	-1
$tRNA^{Gly}$	+	5488-5549	62	GGA			+4
ND3	+	5547-5903	357		ATA	TAG	-3
$tRNA^{Ala}$	+	5902-5966	65	GCA			-2
$tRNA^{Arg}$	+	5967-6030	64	CGA			0
$tRNA^{Asn}$	+	6028-6091	64	AAC			-3
$tRNA^{Ser1}$	+	6089-6154	66	AGC			-3
$tRNA^{Glu}$	+	6155-6217	63	GAA			0
$tRNA^{Phe}$	-	6216-6278	63	TTC			-2
ND5	-	6279-8007	1729		ATG	T	0
$tRNA^{His}$	-	8008-8069	62	CAC			0
ND4	-	8070-9416	1347		ATA	TAA	0
ND4L	-	9410-9706	297		ATG	TAA	-7
$tRNA^{Thr}$	+	9709-9770	62	ACA			+2
$tRNA^{Pro}$	-	9771-9834	64	CCA			0
ND6	+	9840-10355	516		ATT	TAA	+5
Cyt *b*	+	10355-11489	1135		ATG	T	-1
$tRNA^{Ser2}$	+	11490-11556	67	TCA			0
ND1	-	11575-12513	939		ATG	TAA	+18
$tRNA^{Leu1}$	-	12515-12578	64	CTA			+1
16S rRNA	-	12579-13801	1223				0
$tRNA^{Val}$	-	13802-13867	66	GTA			0
12S rRNA	-	13868-14659	792				0
CR	+	14660-15674	1015				0
