# Supplementary material for: Cryptic Species Exist in Vietnamella sinensis Hsu, 1936 (Insecta: Ephemeroptera) from Studies of Complete Mitochondrial Genomes
Source: Insects. 2022 Apr 26;13(5):412. doi: 10.3390/insects13050412 (PMC9143467; doi:10.3390/insects13050412)
Supplement: Supplementary file 1 [file insects-13-00412-s001.zip › TableS4. (QY) location.pdf]

**Table S4.** Location of features in the mtDNA of *V. sinensis* QY.

| Gene | Strand | Position | Length (nuc.) | Anticodon | Start codon | Stop codon | Intergenic nucleotides |
|---|---|---|---|---|---|---|---|
| tRNA$^{Ile}$ | + | 1-65 | 65 | ATC | | | +14 |
| tRNA$^{Gln}$ | - | 80-148 | 69 | CAA | | | +16 |
| tRNA$^{Met}$ | + | 165-228 | 64 | ATG | | | 0 |
| ND2 | + | 229-1251 | 1023 | | ATA | TAA | -2 |
| tRNA$^{Trp}$ | + | 1250-1318 | 69 | TGA | | | -8 |
| tRNA$^{Cys}$ | - | 1311-1371 | 61 | TGC | | | 0 |
| tRNA$^{Tyr}$ | - | 1372-1441 | 70 | TAC | | | -41 |
| COI | + | 1401-2978 | 1578 | | ATA | TAA | -5 |
| tRNA$^{Leu2}$ | + | 2974-3037 | 64 | TTA | | | 0 |
| COII | + | 3038-3725 | 688 | | ATG | T | 0 |
| tRNA$^{Lys}$ | + | 3726-3794 | 69 | AAG | | | 0 |
| tRNA$^{Asp}$ | + | 3795-3861 | 67 | GAC | | | +9 |
| ATP8 | + | 3871-4026 | 156 | | ATA | TAA | -4 |
| ATP6 | + | 4023-4697 | 675 | | ATA | TAA | -1 |
| COIII | + | 4697-5485 | 789 | | ATG | TAA | +4 |
| tRNA$^{Gly}$ | + | 5490-5551 | 62 | GGA | | | -3 |
| ND3 | + | 5549-5905 | 357 | | ATA | TAG | -2 |
| tRNA$^{Ala}$ | + | 5904-5969 | 66 | GCA | | | 0 |
| tRNA$^{Arg}$ | + | 5970-6033 | 64 | CGA | | | -3 |
| tRNA$^{Asn}$ | + | 6031-6094 | 64 | AAC | | | -3 |
| tRNA$^{Ser1}$ | + | 6092-6157 | 66 | AGC | | | 0 |
| tRNA$^{Glu}$ | + | 6158-6220 | 63 | GAA | | | +25 |
| tRNA$^{Phe}$ | - | 6246-6309 | 64 | TTC | | | 0 |
| ND5 | - | 6310-8038 | 1729 | | ATG | T | 0 |
| tRNA$^{His}$ | - | 8039-8100 | 62 | CAC | | | 0 |
| ND4 | - | 8101-9447 | 1347 | | ATG | TAA | -7 |
| ND4L | - | 9441-9737 | 297 | | ATG | TAA | +2 |
| tRNA$^{Thr}$ | + | 9740-9801 | 62 | ACA | | | 0 |
| tRNA$^{Pro}$ | - | 9802-9865 | 64 | CCA | | | +5 |
| ND6 | + | 9871-10386 | 516 | | ATT | TAA | -1 |
| Cyt *b* | + | 10386-11520 | 1135 | | ATG | T | 0 |
| tRNA$^{Ser2}$ | + | 11521-11587 | 67 | TCA | | | +30 |
| ND1 | - | 11618-12556 | 939 | | ATG | TAA | +1 |
| tRNA$^{Leu1}$ | - | 12558-12622 | 65 | CTA | | | 0 |
| 16S rRNA | - | 12623-13843 | 1221 | | | | 0 |
| tRNA$^{Val}$ | - | 13844-13909 | 66 | GTA | | | 0 |
| 12S rRNA | - | 13910-14699 | 790 | | | | 0 |
| CR | | 14700-15610 | 911 | | | | |
