# Supplementary material for: Cryptic Species Exist in Vietnamella sinensis Hsu, 1936 (Insecta: Ephemeroptera) from Studies of Complete Mitochondrial Genomes
Source: Insects. 2022 Apr 26;13(5):412. doi: 10.3390/insects13050412 (PMC9143467; doi:10.3390/insects13050412)
Supplement: Supplementary file 1 [file insects-13-00412-s001.zip › TableS5. RSCU.pdf]

Table S5. Codon numbers and relative synonymous codon usage in the protein coding genes of mt genomes of *V. sinensis* CN/TL (CN/TL), *V. sinensis* QY (QY) and *V. sinensis* (VS) (HM067837).

Codon	Count			RSCU			Codon	Count			RSCU		
	CN/TL	QY	VS (HM067837)	CN/TL	QY	VS (HM067837)		CN/TL	QY	VS (HM067837)	CN/TL	QY	VS (HM067837)
UUU(F)	246	241	250	1.49	1.48	1.5	GCG(A)	14	10	13	0.26	0.18	0.24
UUC(F)	84	85	84	0.51	0.52	0.5	UAU(Y)	133	134	130	1.64	1.65	1.6
UUA(L)	385	364	382	3.74	3.53	3.73	UAC(Y)	29	28	32	0.36	0.35	0.4
UUG(L)	52	57	56	0.5	0.55	0.55	CAU(H)	57	62	58	1.41	1.49	1.43
CUU(L)	95	87	97	0.92	0.84	0.95	CAC(H)	24	21	23	0.59	0.51	0.57
CUC(L)	24	33	20	0.23	0.32	0.2	CAA(Q)	61	52	55	1.65	1.42	1.51
CUA(L)	47	55	49	0.46	0.53	0.48	CAG(Q)	13	21	18	0.35	0.58	0.49
CUG(L)	15	22	11	0.15	0.21	0.11	AAU(N)	106	115	112	1.42	1.53	1.51
AUU(I)	261	237	262	1.79	1.66	1.79	AAC(N)	43	35	36	0.58	0.47	0.49
AUC(I)	30	48	31	0.21	0.34	0.21	AAA(K)	71	64	64	1.54	1.42	1.41
AUA(M)	180	174	180	1.65	1.56	1.65	AAG(K)	21	26	27	0.46	0.58	0.59
AUG(M)	38	49	38	0.35	0.44	0.35	GAU(D)	54	56	58	1.52	1.6	1.66
GUU(V)	89	83	83	1.61	1.49	1.5	GAC(D)	17	14	12	0.48	0.4	0.34
GUC(V)	15	17	16	0.27	0.3	0.29	GAA(E)	61	67	64	1.52	1.68	1.56
GUA(V)	90	95	98	1.63	1.7	1.77	GAG(E)	19	13	18	0.47	0.33	0.44
GUG(V)	27	28	25	0.49	0.5	0.45	UGU(C)	32	29	32	1.83	1.61	1.83
UCU(S)	119	116	117	2.82	2.75	2.79	UGC(C)	3	7	3	0.17	0.39	0.17
UCC(S)	27	33	32	0.64	0.78	0.76	UGA(W)	79	74	76	1.52	1.42	1.46
UCA(S)	44	40	39	1.04	0.95	0.93	UGG(W)	25	30	28	0.48	0.58	0.54
UCG(S)	7	8	8	0.17	0.19	0.19	CGU(R)	19	18	19	1.27	1.22	1.27
CCU(P)	88	74	85	2.32	1.95	2.24	CGC(R)	3	5	3	0.2	0.34	0.2
CCC(P)	29	39	31	0.76	1.03	0.82	CGA(R)	26	24	28	1.73	1.63	1.87
CCA(P)	23	26	24	0.61	0.68	0.63	CGG(R)	12	12	10	0.8	0.81	0.67
CCG(P)	12	13	12	0.32	0.34	0.32	AGU(S)	46	42	49	1.09	0.99	1.17
ACU(T)	102	98	102	2.07	2	2.08	AGC(S)	14	14	10	0.33	0.33	0.24
ACC(T)	17	27	20	0.35	0.55	0.41	AGA(S)	81	84	81	1.92	1.99	1.93
ACA(T)	66	59	70	1.34	1.2	1.43	AGG(S)	0	1	0	0	0.02	0
ACG(T)	12	12	4	0.24	0.24	0.08	GGU(G)	82	68	78	1.36	1.12	1.29
GCU(A)	121	119	117	2.22	2.19	2.15	GGC(G)	12	18	19	0.2	0.3	0.32
GCC(A)	37	37	44	0.68	0.68	0.81	GGA(G)	102	96	99	1.69	1.58	1.64
GCA(A)	46	51	44	0.84	0.94	0.81	GGG(G)	45	61	45	0.75	1	0.75
