# Supplementary material for: Cryptic Species Exist in Vietnamella sinensis Hsu, 1936 (Insecta: Ephemeroptera) from Studies of Complete Mitochondrial Genomes
Source: Insects. 2022 Apr 26;13(5):412. doi: 10.3390/insects13050412 (PMC9143467; doi:10.3390/insects13050412)
Supplement: Supplementary file 1 [file insects-13-00412-s001.zip › TableS1. Tree.pdf]

Table S1. Species information used in the reconstruction of Ephemeroptera phylogeny in this study.

Family	Genus	Species	Length (bp)	GenBank No.	References
Ameletidae	*Ameletus*	*Ameletus* sp. MT-2014	15,141	KM244682	[58]
Baetidae	*Baetis*	*Baetis* sp. PC-2010	14,883	GU936204	unpublished
Baetidae	*Baetis*	*Baetis* sp. ZY-2019	15,391	MT671494	[61]
Baetidae	*Takobia*	*Takobia yixiani*	14,589	GU479735	unpublished
Baetidae	*Cloeon*	*Cloeon dipterum*	15,407	MW149047	unpublished
Caenidae	*Caenis*	*Caenis pycnacantha*	15,351	GQ502451	unpublished
Caenidae	*Caenis*	*Caenis* sp. JYZ-2018	15,254	MG910499	[12]
Caenidae	*Caenis*	*Caenis* sp. JYZ-2020	15,392	MN356096	[60]
Caenidae	*Caenis*	*Caenis* sp. JZ-2021	13,781	MF352163	[62]
Caenidae	unclassified	*Caenidae* sp. Cy2020	15,658	MT671487	[61]
Ephemerellidae	*Ephemerella*	*Ephemerella* sp. MT-2014	14,896	KM244691	[58]
Ephemerellidae	*Ephemerella*	*Ephemerella* sp. Yunnan-2018	15,256	MT274127	[14]
Ephemerellidae	*Serratella*	*Serratella* sp. Liaoning-2019	15,523	MT274128	[14]
Ephemerellidae	*Serratella*	*Serratella* sp. Yunnan-2018	15,134	MT274129	[14]
Ephemerellidae	*Serratella*	*Serratella zapekinae*	15,703	MT274130	[14]
Ephemerellidae	*Serratella*	*Serratella* sp. JZ-2021	16,751	MF352164	[62]
Ephemerellidae	*Torleya*	*Torleya mikhaili*	15,042	MT535766	[41]
Ephemerellidae	*Torleya*	*Torleya nepalica*	15,599	MT274132	[14]
Ephemerellidae	*Torleya*	*Torleya grandiforceps*	15,330	MT274131	[14]
Ephemerellidae	*Drunella*	*Drunella* sp. JZ-2021	15,128	MF352150	[62]
Ephemerellidae	*unclassified*	*Ephemerellidae* sp. JZ-2021	15,154	MF352168	[62]
Ephemerellidae	*Uracanthella*	*Uracanthella* sp. JZ-2021	15,160	MF352162	[62]
Ephemeridae	*Ephemera*	*Ephemera rufomaculata*	14,451	MF352156	[62]
Ephemeridae	*Ephemera*	*Ephemera shengmi*	15,149	MF352161	[62]
Ephemeridae	*Ephemera*	*Ephemera. orientalis*	16,463	EU591678	[56]
Ephemeridae	*Ephemera*	*Ephemera* sp. XL-2019	15,314	MK951659	unpublished
Heptageniidae	*Afronurus*	*Afronurus rubromaculata*	15,519	MK642294	[8]
Heptageniidae	*Afronurus*	*Afronurus yixingensis*	15,883	MK642297	[8]
Heptageniidae	*Cinygmina*	*Cinygmina* sp. 07BF85	15,473	MW450876	[8]
Heptageniidae	*Cinygmina*	*Cinygmina* sp. 07BF86	15,696	MW450877	[8]
Heptageniidae	*Cinygmina*	*Cinygmina* sp. 07BF96	15,491	MW450878	[8]
Heptageniidae	*Cinygmina*	*Cinygmina* sp.1 YW01BF06	15,360	MK642295	[8]
Heptageniidae	*Cinygmina*	*Cinygmina* sp.2 CLS53BF04	15,866	MK642296	[8]
Heptageniidae	*Cinygmina*	*Cinygmina furcata*	15,420	MK642293	[8]
Heptageniidae	*Cinygmina*	*Cinygmina obliquistriata*	14,964	MF352149	[62]
Heptageniidae	*Epeorus*	*Epeorus* sp. *'Iron'* JZ-2021	15,508	MF352155	[62]
Heptageniidae	*Epeorus*	*Epeoru herklotsi*	15,502	MG870104	[45]

Heptageniidae	*Epeorus*	*Epeorus* sp. JZ-2014	15,338	KJ493406	unpublished
Heptageniidae	*Epeorus*	*Epeorus* sp. MT-2014	15,456	KM244708	[58]
Heptageniidae	*Epeorus*	*Epeorus dayongensis*	15,337	MK642298	[8]
Heptageniidae	*Epeorus*	*Epeorus* sp. LA03FY06	15,514	MK642299	[8]
Heptageniidae	*Paegniodes*	*Paegniodes cupulatus*	15,715	HM004123	unpublished
Heptageniidae	*Parafronurus*	*Parafronurus youi*	15,481	EU349015	[55]
Heptageniidae	*Stenacron*	*Stenacron interpunctatum*	15,330	MK642305	[8]
Heptageniidae	*Stenonema*	*Stenonema femoratum*	15,332	MK642306	[8]
Heptageniidae	*Maccaffertium*	*Maccaffertium vicarium*	15,324	MK642304	[8]
Heptageniidae	*Maccaffertium*	*Maccaffertium mediopunctatum*	15,324	MK642303	[8]
Heptageniidae	*Heptagenia*	*Heptagenia* sp. JZ-2021	15,352	MF352153	[62]
Heptageniidae	*Leucrocuta*	*Leucrocuta aphrodite*	15,428	MK642301	[8]
Heptageniidae	unclassified	*Heptageniidae* sp. YW03BF02	15,663	MK642300	[8]
Isonychiidae	*Isonychia*	*Isonychia ignota*	15,105	HM143892	unpublished
Isonychiidae	*Isonychia*	*Isonychia kiangsinensis*	15,456	MH119135	[9]
Isonychiidae	*Isonychia*	*Isonychia* sp. XL-2019	15,618	MK951658	[59]
Isonychiidae	*Isonychia*	*Isonychia ignota*	15,105	MF352147	unpublished
Leptophlebiidae	*Choroterpides*	*Choroterpides apiculata*	15,199	MN807287	[13]
Leptophlebiidae	*Habrophlebiodes*	*Habrophlebiodes zijinensis*	14,355	GU936203	unpublished
Leptophlebiidae	*Leptophlebia*	*Leptophlebia* sp. JZ-2021	15,534	MF352160	[62]
Potamanthidae	*Potamanthus*	*Potamanthus* sp. MT-2014	14,937	KM244674	[58]
Potamanthidae	*Potamanthus*	*Rhoenanthus* sp. JZ-2021	14,118	MF352145	[62]
Potamanthidae	*Potamanthus*	*Potamanthus kwangsiensis*	13,988	MF352158	[62]
Polymitarcyidae	*Ephoron*	*Ephoron yunnanensis*	13,949	MF352159	[62]
Siphlonuridae	*Siphlonurus*	*Siphlonurus aestivalis*	15,120	MT862395	unpublished
Siphlonuridae	*Siphlonurus*	*Siphlonurus* sp. MT-2014	14,745	KM244684	[58]
Siphluriscidae	*Siphluriscus*	*Siphluriscus chinensis*	16,616	HQ875717	[57]
Siphluriscidae	*Siphluriscus*	*Siphluriscus chinensis*	14,424	MF352165	[62]
Teloganodidae	unclassified	*Teloganodidae* sp. MT-2014	12,435	KM244703	[58]
Teloganodidae	unclassified	*Teloganodidae* sp. MT-2014	2,817	KM244670	[58]
Vietnamellidae	*Vietnamella*	*Vietnamella* sp. MT-2014	15,043	KM244655	[58]
Vietnamellidae	*Vietnamella*	*Vietnamella dabieshanensis*	15,761	HM067837	unpublished
Vietnamellidae	*Vietnamella*	*Vietnamella* sp. JZ-2021	16,006	MF352146	[62]
Vietnamellidae	*Vietnamella*	*Vietnamella sinensis* TL	15,674	OK265109	this study
Vietnamellidae	*Vietnamella*	*Vietnamella sinensis* QY	15,610	OK265110	this study
Vietnamellidae	*Vietnamella*	*Vietnamella sinensis* CN	15,674	OK265111	this study
